# Identification and characterization of the kynurenine pathway in the pond snail *Lymnaea stagnalis*

**DOI:** 10.1038/s41598-022-19652-0

**Published:** 2022-09-16

**Authors:** Benatti Cristina, Rivi Veronica, Alboni Silvia, Grilli Andrea, Castellano Sara, Pani Luca, Brunello Nicoletta, Blom Johanna M.C., Bicciato Silvio, Tascedda Fabio

**Affiliations:** 1 Department of Life Sciences, University of Modena and Reggio Emilia, Via Campi, 287, 41125 Modena, Italy; 2 Department of Biomedical, Metabolic and Neural Sciences, University of Modena and Reggio Emilia, Modena, Italy; 3 Centre of Neuroscience and Neurotechnology, University of Modena and Reggio Emilia, Modena, Italy; 4 CIB, Consorzio Interuniversitario Biotecnologie, Trieste, Italy; 5 Biomage Ltd, Edinburgh, EH4 2HS UK; 6 Department of Psychiatry and Behavioral Sciences, University of Miami, Miami, USA

**Keywords:** Preclinical research, Translational research, Sequence annotation

## Abstract

Dysregulation of the kynurenine pathway (KP) is implicated in many human diseases and disorders, from immunological, metabolic, neurodegenerative, and neuropsychiatric conditions to cancer, and represents an appealing target for new therapeutic approaches. In this intricate scenario, invertebrates, *like Lymnaea stagnalis* (LS), provide a flexible tool to unravel the complexity of the KP. Starting from the available *LS* genome and transcriptome, we identified putative transcripts of all KP enzymes containing an ORF; each predicted protein possessed a high degree of sequence conservation to known orthologues of other invertebrate and vertebrate model organisms. Sequences were confirmed by qualitative PCR and sequencing. At the same time, the qRT-PCR analysis revealed that Lym IDO-like, Lym TDO-like, Lym AFMID-like, Lym KMO-like, Lym AADAT-like, Lym KYAT I/III-like, Lym KYNU-like, Lym HAAO-like, and Lym ACMSD-like showed widespread tissue expression. Then, tryptophan, kynurenine, kynurenic acid, anthranilic acid, 3-hydroxy-kynurenine, xanthurenic acid, picolinic acid, and quinolinic acid were identified in the hemolymph of LS by UHPLC-Q exactive mass spectrometer. Our study provides the most thorough characterization to date of the KP in an invertebrate model, supporting the value of LS for future functional studies of this pathway at the cellular, synaptic, and behavioral levels.

## Introduction

**Figure 1:**
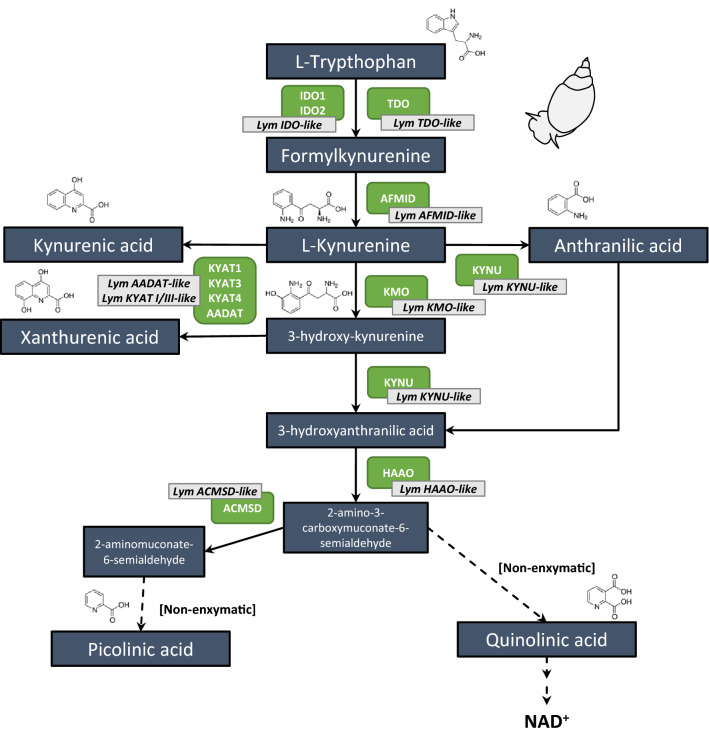
Predicted kynurenine pathway in *Lymnaea stagnalis*. *IDO* Indoleamine-2,3-dioxygenase, *TDO* Tryptophan-2,3-dioxygenase, *AFMID* Kynurenine formamidase, *AADAT* Aminoadipate aminotransferase, *KYAT* Kynurenine-oxoglutarate transaminase, *KMO* Kynurenine 3-monooxygenase, *KYNU* Kynureninase, *HAAO*  3-hydroxyanthranilate 3,4-dioxygenase, *ACMSD* 2-amino-3-carboxymuconate-6-semialdehyde decarboxylase, *NAD* Nicotinamide-Adenine-Dinucleotide.

The kynurenine pathway (KP) is crucial for peripheral and central catabolism of L-tryptophan (L-TRP), the essential amino acid precursor of serotonin and melatonin. The KP, a complex multi-step cascade with biologically active metabolites, catabolizes more than 95% of dietary tryptophan ^1^ (Fig. 1).

Physiologically, it leads to the production of the redox cofactor oxidized nicotinamide adenine dinucleotide (NAD^+^) and contributes to the establishment and maintenance of the immune privilege in several sites of the organism (including the brain, eye, and colon) ^2^. Moreover, many kynurenines have neuro-modulatory properties, regulating neuroplasticity and neurotoxicity ^3^, such as the N-methyl-d-aspartate (NMDA) receptor agonist quinolinic acid (QUINA), the NMDA antagonist kynurenic acid (KYNA), and the neuroactive 3-hydroxy-kynurenine (3HK) and the immunosuppressive 3-hydroxyanthranilic acid (3HANA) ^2^.

The kynurenine pathway starts with the oxidative cleavage of TRP by tryptophan 2,3-dioxygenase (TDO), which in humans and rodents is mainly expressed in the liver and brain, or by indoleamine-2,3-dioxygenase (IDO). Both these enzymes convert TRP to N-formylkynurenine ^4,5^, which is then metabolized by the enzyme kynurenine formamidase (AFMID) into the pivotal KP intermediate L-kynurenine (KYN). KYN in turn can be metabolized by at least three different enzymes: i) kynurenine 3-monooxygenase (KMO), forming 3-hydroxy-kynurenine; ii) four kynurenine aminotransferase (KYAT I-IV) isoenzymes that catalyze the transamination of KYN into KYNA; and iii) kynureninase (KYNU), developing anthranilic acid (ANA) ^1^ (Fig. 1).

Under normal conditions, the KMO branch seems to be the major metabolic route in the KP since this enzyme has the highest affinity for KYN, while metabolism via the KYNU branch only occurs when KYN concentrations are elevated ^2–4^. Continuing along the KMO-branch, 3-hydroxy-kynurenine is metabolized by KYNU into 3HANA, which has a 20-fold higher affinity for 3HK than for KYN, favoring the generation of 3HANA over ANA. 3-hydroxyanthranilate 3,4-dioxygenase (HAAO) then catalyzes the conversion of 3HANA to an unstable intermediate, 2-amino-3-carboxymuconic semialdehyde, which is then preferentially converted into QUINA by non-enzymatic cyclization ^6^. The balance between the excitotoxin, quinolinic acid, and neuroprotective picolinic acid (PICA) is maintained by the enzyme 2-amino 3-carboxymuconate 6-semialdehyde decarboxylase (ACMSD). QUINA is the endogenous source of nicotinamide and NAD^+^^3^.

The relative balance of the KP metabolites generated in peripheral tissues and the central nervous system (CNS) appears to be regulated at different interdependent levels ^2^. Moreover, several lines of evidence propose an essential role of this pathway in physiological and pathological conditions. Alterations and imbalances in the rheostat of neuroactive KP metabolites have been implied in a wide range of diseases and disorders, including inflammation/immune disorders, endocrine/metabolic conditions, Alzheimer’s disease, amyotrophic lateral sclerosis, Huntington’s disease, cancer, depression, and schizophrenia ^7–10^.

Thus, the KP has become an appealing target for developing new therapeutic approaches. Unfortunately, up to date, pharmacological inhibitors of different KP enzymes have led to mixed results in both preclinical and clinical studies ^11^.

In this complex scenario, invertebrates represent a flexible tool to study fundamental and conserved mechanisms of CNS physiology and pathology, such as the kynurenine pathway ^12,13^. The kynurenine metabolism is highly conserved throughout the eukaryotic lineage from yeast to humans ^1^. Until now, the most common organisms used to investigate the KP during immune activation and different human conditions have been primates and rodents.

Research using invertebrate models has been associated with significant experimental efficiency due to the reduced time needed for experiments and the low costs required for animal care thanks to their short generation times, numerous offspring, and the fact that they can be more easily manipulated experimentally. While many studies and breakthroughs in our understanding of neurodegenerative diseases have used *D. melanogaster* and *C. elegans,* their role in the study of the KP pathway has not been established yet. In fact, while TDO has been identified in both *C. elegans* and *D. melanogaster*, IDO has not been detected in either species ^5^. Furthermore, no IDO genes have been found in Arthropods yet, whereas some species of Nematodes (e.g., *Brugia malayi* and *Necator americanus*) have proven to carry the IDO gene, no corresponding gene was found in *C. elegans* ^5,14–16^.

Although invertebrates can never replace mammal models in preclinical studies, a full characterization of the KP in one of these models may be beneficial to unraveling its complexity while translating results to mammals. An interesting candidate model system is the gastropod mollusk *Lymnaea stagnalis (L. stagnalis*, Linnaeus, 1758), which has a relatively long average life span and, like other gastropods such as *Aplysia californica, Biomphalaria glabrata*, and *Pomacea canaliculata* ^17^, has proven to be a valuable tool in gaining a deeper understanding of the functioning of the nervous system. *L. stagnalis* belongs to the *phylum* Mollusca class Gastropoda and has been widely used to investigate genetic and epigenetic processes ^18–20^, aging ^21,22^, learning and memory ^23–25^, and for ecotoxicological studies ^26,27^. The CNS of *L. stagnalis* consists of approximately 20,000 readily identifiable neurons, organized in a ring of interconnected ganglia, offering a relatively large amount of biological material that could be molecularly, physiologically, and morphologically analyzed ^13^.

Considering the importance of the kynurenine pathway in different fields from neuroscience to immunology and pharmacology and the different fields in which our versatile model has been and could be adopted, in this study we aimed to identify and characterize the kynurenine pathway in *L. stagnalis.* Starting from the *L. stagnalis* genome and transcriptional profiles, we first identified and annotated putative transcripts coding for the KP enzymes in the CNS of *L. stagnalis*. Then, we identified the KP metabolites in the hemolymph of *L. stagnalis* using a UHPLC-Q exactive mass spectrometer, one of the most reliable techniques for metabolite identification and characterization.

Overall, our results indicate that *L. stagnalis* may represent an appropriate model organism to study the involvement of the kynurenine pathway in neuropsychiatric disorders and immune diseases and further corroborate its use as a model for translational neuroscience.

## Results

### Identification and characterization of putative transcripts of the kynurenine pathway in *L. stagnalis*

To identify putative KP genes in *L. stagnalis,* we first aligned with *BLASTx* all 328,378 contigs of *L. stagnalis* genome to 36,675 protein sequences annotated with RefSeq ID of *B. glabrata*, i.e., one of the *L. stagnalis* phylogenetically closest organisms for which annotated proteins are available, and to 76,216 and 110,386 annotated protein sequences of *M. musculus* and *H. sapiens*, respectively. At an E-value cutoff of 1E-6, *BLASTx* returned 81,845 contigs having a hit in *B. glabrata*, 30,478 contigs having a hit in *M. musculus*, and 30,001 contigs having a hit in *H. sapiens.* Using the RefSeq ID of protein sequences, we annotated contigs with at least one hit with the *B. glabrata*, mouse, and human gene symbols and with Gene Ontology (GO) and KEGG pathway terms (Supplementary Table 1). We quantified contigs’ gene expression levels using publicly available raw RNA-sequencing (RNA-seq) data from *L. stagnalis* central nervous system ^18^. We assembled the transcriptome with Cufflinks aligning the RNA-seq reads to the *L. stagnalis* genome with TopHat. Before assembly, we trimmed the original 81,851,004 reads and filtered them for quality obtaining 78,245,030 reads, 64.1% of which (50,124,678 reads) were mapped to contigs by TopHat. The gene expression level of each contig was then quantified, converting into TPM the counts of 44,576,148 uniquely mapping reads (see Methods for details; Supplementary Table 1).

**Figure 2:**
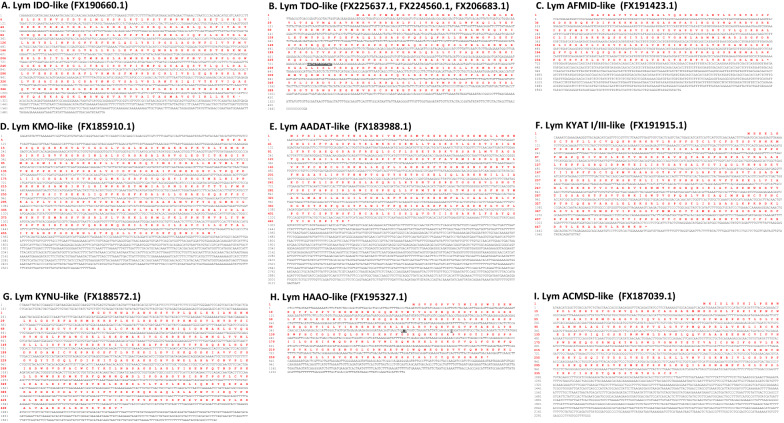
Nucleotide and deduced amino acid sequence of the putative enzymes of the KP pathway. (**A**) Lym IDO-like, (**B**) Lym TDO-like, (**C**) Lym AFMID-like, (**D**) Lym KMO-like, (**E**) Lym AADAT-like, (**F**) Lym KYAT I/III-like, (**G**) Lym KYNU-like, (**H**) Lym HAAO-like, (**I**) Lym ACMSD-like. Differences between the amplified and the deposited sequences are bold and underlined.

**Figure 3:**
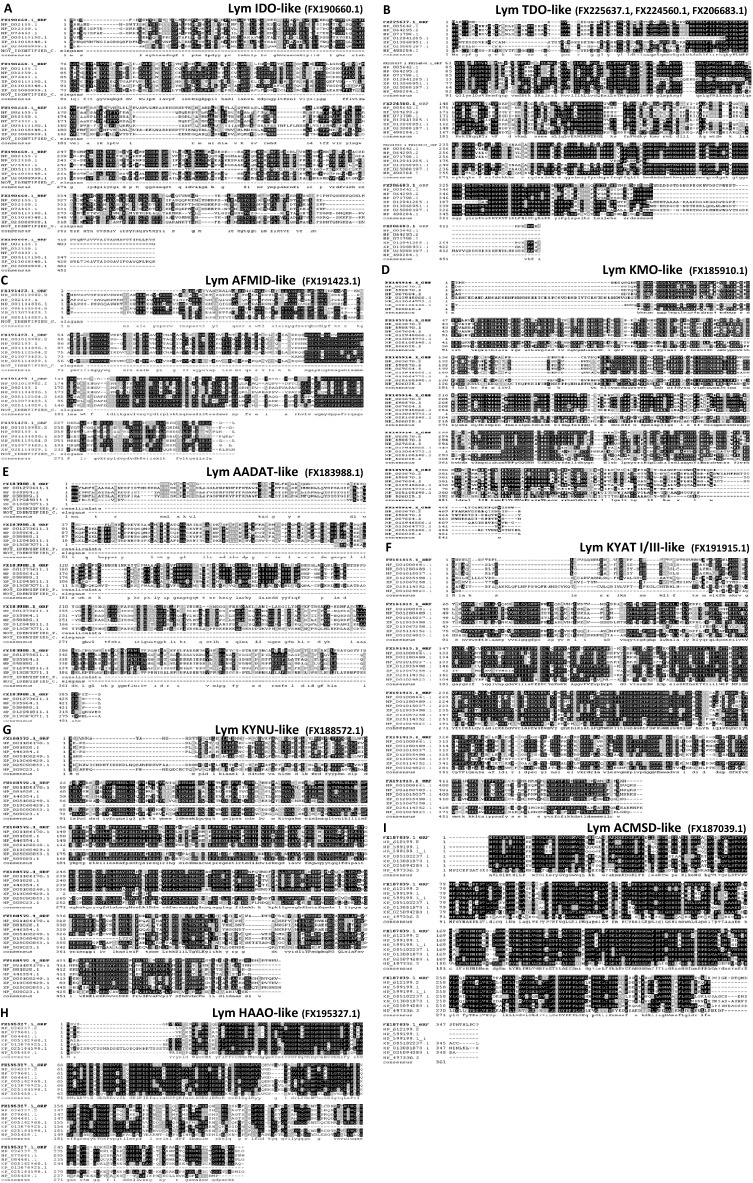
Multiple sequence alignments of each putative KP enzyme in *L. stagnalis* with the amino acid sequences of the corresponding enzyme from *H. sapiens*, *M. musculus*, *R. norvegicus*, *A. californica*, *B. glabrata*, *P. canaliculata*, and *C. elegans*. (**A**) Lym IDO-like, (**B**) Lym TDO-like, (**C**) Lym AFMID-like, (**D**) Lym KMO-like, (**E**) Lym AADAT-like, (**F**) Lym KYAT I/III-like, (**G**) Lym KYNU-like, (**H**) Lym HAAO-like, (**I**) Lym ACMSD-like. Box shade alignment displays show conserved residues (identical black, similar grey).

**Figure 4:**
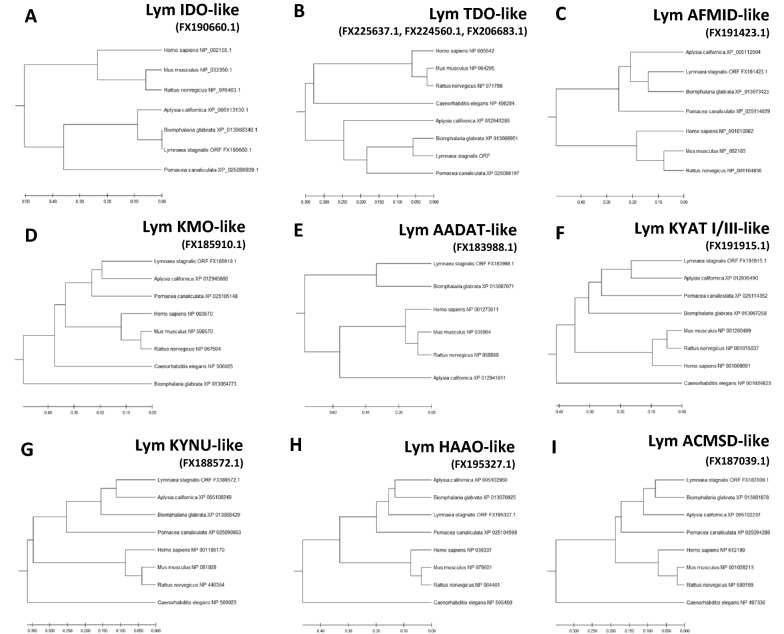
Molecular phylogenetic tree of the putative KP enzymes in *L. stagnalis*. (**A**) Lym IDO-like, (**B**) Lym TDO-like, (**C**) Lym AFMID-like, (**D**) Lym KMO-like, (**E**) Lym AADAT-like, (**F**) Lym KYAT I/III-like, (**G**) Lym KYNU-like, (**H**) Lym HAAO-like, (**I**) Lym ACMSD-like. The tree is drawn to scale, with branch lengths in the same units as the evolutionary distances used to infer the phylogenetic tree. The evolutionary distances were computed using the Poisson correction method and are in the units of the number of amino acid substitutions per site. This analysis involved 7–8 amino acid sequences. Evolutionary analyses were conducted in MEGA X. The GenBank accession numbers of proteins used are indicated and listed in Table 1.

Using the contig annotation table, we identified 20 *L. stagnalis* contigs annotated to the nine genes of the KP pathway in *B. glabrata*, with the IDO-like orthologue and the two putative isoforms of aminotransferases named as myoglobin-like, kynurenine-oxoglutarate transaminase 1/3-like, and 2-aminoadipate transaminase-like, respectively (Supplementary Table 2). To identify transcripts in these contigs, we first manually extracted the putative exonic sequences visualizing the RNA-seq reads with the Integrative Genomics Viewer (IGV). Then, we retrieved the precise mRNA sequence aligning these putative exonic sequences to the transcriptome shotgun assembly of *L. stagnalis*. This procedure allowed the identification of one specific transcript per each KP enzyme, except for tryptophan 2,3-dioxygenase-like, which was associated with three small transcripts (FX225637.1, FX224560.1, and FX206683.1; Supplementary Table 2). All transcripts resulted to be expressed (TPM > 0) in the publicly available *L. stagnalis* CNS transcriptome. To confirm whether these transcripts were expressed in the *L. stagnalis* CNS, we performed qPCR on each transcript using the set of primers listed in Supplementary Table 3 and displayed in Supplementary Fig. 1. The PCR products were purified, sequenced, and aligned to FX190660.1 (Lym IDO-like), FX191423.1 (Lym AFMID-like), FX185910.1 (Lym KMO-like), FX183988.1 (Lym AADAT-like), FX191915.1 (Lym KYAT-like), FX188572.1 (Lym KYNU-like), and FX187039.1 (Lym ACMSD-like). All sequences, except for Lym TDO-like and Lym HAAO-like, uniquely matched their respective templates. For Lym TDO-like, we used a combination of primers on FX225637.1, FX224560.1, and FX206683.1 and amplified and sequenced a single PCR product resulting from the variety of the three transcripts with a 15 bp gap (TATTCCTCTATAAGG) between the first two (Supplementary Fig. 1). Moreover, the amplified sequence of Lym HAAO-like missed an A in position 422 and presented a T to C mutation in position 447 (Supplementary Fig. 1). All transcripts contained at least one ORF (Fig. 2), whose identity was further confirmed by aligning its sequence with the amino acid sequences of the corresponding enzyme from *H. sapiens*, *M. musculus*, *R. norvegicus*, *A. californica*, *B. glabrata*, *P. canaliculata*, and *C. elegans* ^28^ (Table 1). In *C. elegans*, we identified six orthologs that had an average homology of about 48% with the corresponding sequence of *L. stagnalis*. A similar degree of homology was found with mammalian sequences, while we observed a higher degree of homology with mollusk sequences (i.e., 63% for *P. canaliculata*, 64% for *A. californica*, and 70% for *B. glabrata)*. AADAT and ACMSD were the less (30% average homology) and the most (average homology of 71%) conserved genes, respectively (Table 1). This conservation pattern was also confirmed using multiple sequence alignment between each putative enzyme identified in *L. stagnalis* and orthologues from other species (Fig. 3). Phylogenetic analyses revealed a predictable pattern in the relatedness of *L. stagnalis* sequences to those of other *Mollusca* (Fig. 4). Finally, the PFAM analysis confirmed that all the KP-predicted enzymes had the expected domains (Supplementary Fig. S2).

**Table 1:** Homology between the ORF of the putative KP enzymes of *L. stagnalis* with orthologues from different organisms.

Enzyme	*Lymnaea stagnalis*		*Homo sapiens*				*Mus musculus*			*Rattus norvegicus*			*Aplysia californica*			*Biomphalaria glabrata*			*Pomacea canaliculata*			*Caenorhabditis elegans*		
	Contig	Transcript	Predicted size ORF (aa)	Ortholog	Identities	E-value BLASTp	Ortholog	Identities	E-value BLASTp	Ortholog	Identities	E-value BLASTp	Ortholog	Identities	E-value BLASTp	Ortholog	Identities	E-value BLASTp	Ortholog	Identities	E-value BLASTp	Ortholog	Identities	E-value BLASTp
Lym IDO-like	FCFB01037212.1 FCFB01037213.1 FCFB01037214.1	FX190660.1	443	NP_002155.1	152/412 (37%)	1.0E-84	NP_032350.1	147/407 (36%)	1.0E-84	NP_076463.1	154/408 (38%)	8.0E-90	XP_005113130.1	74/211 (35%)	1.0E-31	XP_013068348.1	262/443 (59%)	0.0E + 00	XP_025080939.1	191/404 (47%)	5E-140	–	–	–
Lym TDO-like	FCFB01109750.1 FCFB01109751.1 FCFB01255639.1 FCFB01297038.1	FX224560.1 FX206683.1 FX225637.1	418	NP_005642.1	198/380 (52%)	4.0E-133	NP_064295.2	185/354 (52%)	4.0E-127	NP_071798.1	189/355 (53%)	5.0E-130	XP_012941285.1	117/170 (69%)	7.0E-74	XP_013068951.1	339/419 (81%)	0.0E + 00	XP_025088197.1	264/398 (66%)	0.0E + 00	NP_498284.1	200/370 (54%)	9.0E-135
Lym AFMID-like	FCFB01077058.1 FCFB01161380.1 FCFB01232167.1	FX191423.1	272	NP_001010982.2	95/242 (39%)	6.0E-53	NP_082103.1	98/270 (36%)	1.0E-49	NP_001104836.1	99/274 (36%)	2.0E-48	XP_005112504.2	178/275 (65%)	6.0E-138	XP_013073423	115/155 (74%)	1.0E-80	XP_025114029.1	160/277 (58%)	2.0E-119	–	–	–
Lym KMO-like	FCFB01020652.1	FX185910.1	479	NP_003670.2	238/437 (54%)	0.0E + 00	NP_598570.1	249/440 (57%)	0.0E + 00	NP_067604.1	235/417 (56%)	5.0E-175	XP_012945866.1	324/464 (70%)	0.0E + 00	XP_013064773.1	59/138 (43%)	2.0E-35	XP_025105148.1	280/437 (64%)	0.0E + 00	NP_506025.1	225/460 (49%)	2.0E-156
Lym AADAT-like	FCFB01016053.1	FX183988.1	450	NP_001273611.1	84/329 (26%)	9.0E-28	NP_035964.1	98/405 (24%)	3.0E-19	NP_058889.1	88/334 (26%)	1.0E-22	XP_012941811.1	78/334 (23%)	4.0E-14	XP_013087071.1	138/279 (49%)	1.0E-87	–	–	–	–	–	–
Lym KYAT-like	FCFB01033100.1	FX191915.1	462	NP_001008661.1	234/442 (53%)	1.0E-175	NP_001280489.1	236/436 (54%)	2.0E-168	NP_001015037.1	231/444 (52%)	2.0E-167	XP_012935490.1	323/425 (76%)	0.0E + 00	XP_013067258.1	144/184 (78%)	8.0E-109	XP_025114352.1	280/451 (62%)	0.0E + 00	NP_001024823.1	187/412 (45%)	2.0E-133
Lym KYNU-like	FCFB01070793.1	FX188572.1	485	NP_001186170.1	247/461 (54%)	5.0E-177	NP_081828.1	243/463 (52%)	4E-172	NP_446354.1	247/455 (54%)	4.0E-171	XP_005108249.1	379/470 (81%)	0.0E + 00	XP_013068429.1	107/132 (81%)	9.0E-76	XP_025090853.1	311/482 (65%)	0.0E + 00	NP_509023.1	243/454 (54%)	7.0E-175
Lym ACMSD-like	FCFB01196791.1 FCFB01209656.1 FCFB01252153.1 FCFB01272611.1 FCFB01319919.1	FX187039.1	355	NP_612199.2	232/331 (70%)	0.0E + 00	NP_001028213.1	236/336 (70%)	0.0E + 00	NP_599199.1	235/336 (70%)	0.0E + 00	XP_005102237.1	282/333 (85%)	0.0E + 00	XP_013081878.1	301/352 (86%)	0.0E + 00	XP_025094288.1	249/349 (71%)	0.0E + 00	NP_001022935.1	167/383 (44%)	2.0E-106
Lym HAAO-like	FCFB01141163.1	FX195327.1	293	NP_036337.2	143/283 (51%)	3.0E-111	NP_079601.1	143/284 (50%)	2.0E-100	NP_064461.1	143/284 (50%)	3.0E-102	XP_005102960.1	210/289 (73%)	5.0E-170	XP_013070925.1	167/221 (76%)	4.0E-130	XP_025104598.1	201/291 (69%)	1.0E-157	NP_505450.1	111/270 (41%)	3.0E-62

The size of open reading frame (ORF) of the KP enzymes in *L. stagnalis* and the corresponding orthologues from *H. sapiens*, *M. musculus*, *R. norvegicus*, *A. californica*, *B. glabrata*, *P. canaliculata*, and *C. elegans* are reported*.* The identity and the E-value are indicated for each target.

### Primer specificity, efficiency, and validation

qRT-PCR was used to evaluate the expression levels of the identified *L. stagnalis* KP-enzymes. All gene-specific primers for the analysis are shown in Supplementary Table S3. All primers produced consistent results without amplifying off-target products or generating primer dimers. Following amplification, each primer pair produced amplicons that yielded single bands at the correct size after electrophoresis in 2% agarose gels (Supplementary Fig. S3). Additionally, no amplification was observed in cDNA from another pulmonated gastropod *P. canaliculata* or controls that lacked reverse transcriptase in the RT-PCR. Primer specificity was also checked by melt curve analysis. A single sharp peak with no primer-dimer was observed for all used primer pairs (Supplementary Fig. S4).

Results showed that the PCR efficiency was between 94.82 and 125.30%, and the R^2^ of primers was greater than 0.981. In addition, the mean Cq of all KP-enzymes ranged from 25.41 to 30.02 for 20 ng of cDNA with moderately abundant mRNA levels in the ganglia of *L. stagnalis* (Supplementary Table S3).

### Comparison of expression levels of KP-like enzymes among several tissues of *L. stagnalis*

**Figure 5:**
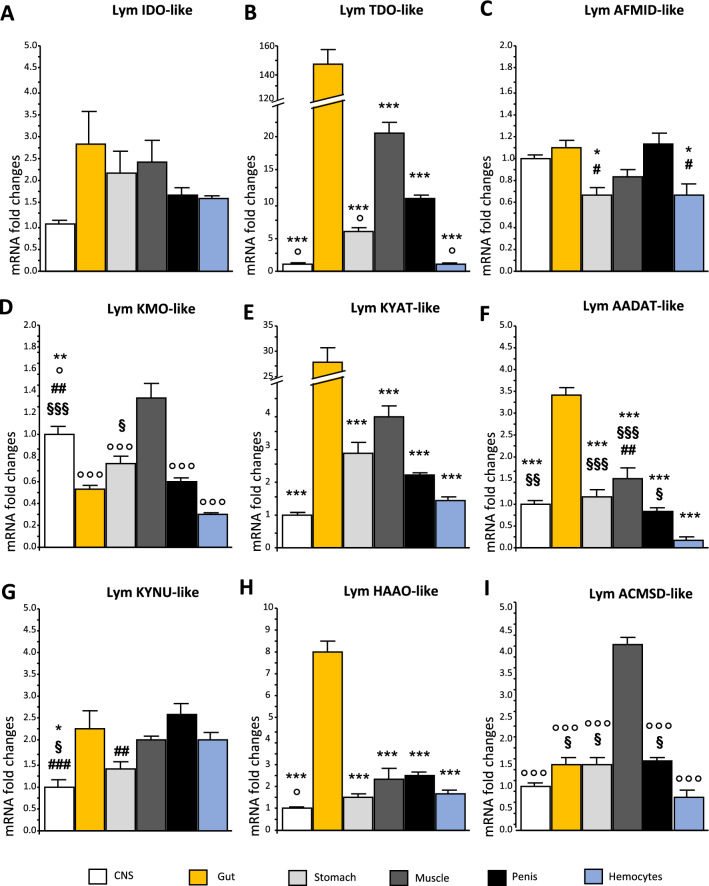
Comparison of mRNA expression levels of putative KP enzymes in different tissues of *L. stagnalis*. (**A**) Lym IDO-like, (**B**) Lym TDO-like, (**C**) Lym AFMID-like, (**D**) Lym KMO-like, (**E**) Lym AADAT-like, (**F**) Lym KYAT I/III-like, (**G**) Lym KYNU-like, (**H**) Lym HAAO-like, (**I**) Lym ACMSD-like mRNA expression levels were analyzed in snail CNS (white bars), gut (yellow bars), stomach (light grey bars), foot (i.e., muscle—dark grey bars), penis (black bars), and hemocytes (light-blue bars). Relative mRNA expression levels were determined by qRT-PCR (2^−ΔΔCt^), with the geometric mean of the reference genes (Lym TUB and Lym EF1a) as endogenous control and CNS as calibrator. *N* = 4 pools of 4 snails for each tissue. Data are expressed as means ± S.E.M and were analyzed with one-way ANOVA followed by Tukey's post hoc test: ***p < 0.001, **p < 0.01, *p < 0.05 versus gut; °°°*p* < 0.001, °°*p* < 0.01, °*p* < 0.05 versus muscle; ###*p* < 0.001, ##*p* < 0.01, #*p* < 0.05 versus penis; §§§*p* < 0.001, §§*p* < 0.01, §*p* < 0.05 versus hemocytes.

We next evaluated the levels of *L. stagnalis* KP transcripts in the CNS and other tissues of the snail, such as the gut (i.e., digestive gland), stomach, muscle (foot), penis, and hemocytes (*n* = 4 pools of 4 snails per each tissue). All KP-enzymes were ubiquitously transcribed in the *L. stagnalis* tissues with different relative abundance patterns (Fig. 5). Specifically, we found that Lym TDO-like was expressed at higher levels in the gut with respect to all the other tissues [F(5; 23) = 175.671; *p* < 0.0001]. Tukey’s post hoc test also revealed that the levels of Lym TDO-like mRNA in the muscle were higher than in SNC, stomach, and hemocytes. In contrast, Lym IDO-like was not differentially expressed in the considered tissues [F(5; 23) = 2.510; *p* = 0.068 with one-way ANOVA]. Lym AFMID-like mRNA levels in the gut and penis were significantly higher than in the stomach and hemocytes. No other difference was detected in any other tissue [F(5; 23) = 6.063; *p* = 0.002). The expression levels of Lym KMO-like in the CNS were significantly higher than those in the penis, gut, muscle, and hemocytes [F(5;23) = 29.639; *p* < 0.001]. Moreover, post-hoc analyses showed that the expression of this gene was significantly higher in the muscle than in all other tissues, while mRNA levels in the stomach were significantly higher than those in the hemocytes. The expression of Lym HAAO-like and Lym KYAT-like was significantly higher in the gut with respect to all the other tissues [F(5;23) = 66.715; p < 0.0001 and F(5;23) = 91.652; *p* < 0.0001, respectively]. We also found that the expression levels of Lym AADAT-like were significantly higher in the gut than in all other tissues considered [F(5;23) = 70.261; *p* < 0.0001]. Post-hoc analyses revealed that the mRNA levels of this gene in the hemocytes were significantly lower with respect to all the other areas, and its expression in the penis was significantly lower than in the muscle. The mRNA levels of Lym KYNU-like in the CNS were significantly lower compared to those in the penis, gut, and hemocytes [F(5;23) = 6.923; *p* < 0.001], and its expression in the stomach was significantly lower than that in the penis. Finally, we found that the mRNA levels of Lym ACMSD-like were significantly higher in the muscle with respect to all the other tissues [F(5;23) = 70.850; *p* < 0.0001]. Post-hoc tests also revealed that the expression levels of this gene in the hemocytes were significantly lower than that in the penis, gut, stomach, and muscle.

### Identification of KP metabolites in the hemolymph.

**Figure 6:**
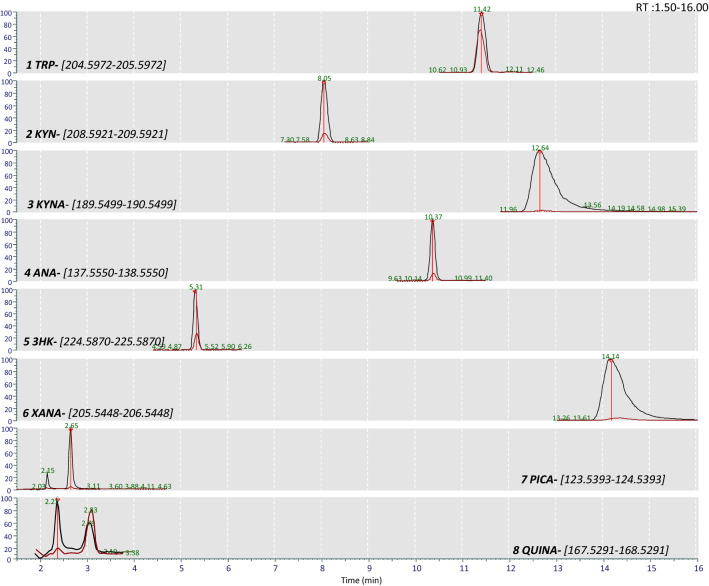
Extracted ion chromatogram of Tryptophan (Trp), kynurenine (KYN), kynurenic acid (KYNA), anthranilic acid (ANA), 3-hydroxy-kynurenine (3HK), xanthurenic acid (XANA), picolinic acid (PICA), and quinolinic acid (QUINA) in hemolymph (red) or hemolymph spiked with a mixture of internal standard (black). *RT*: retention time.

**Figure 7:**
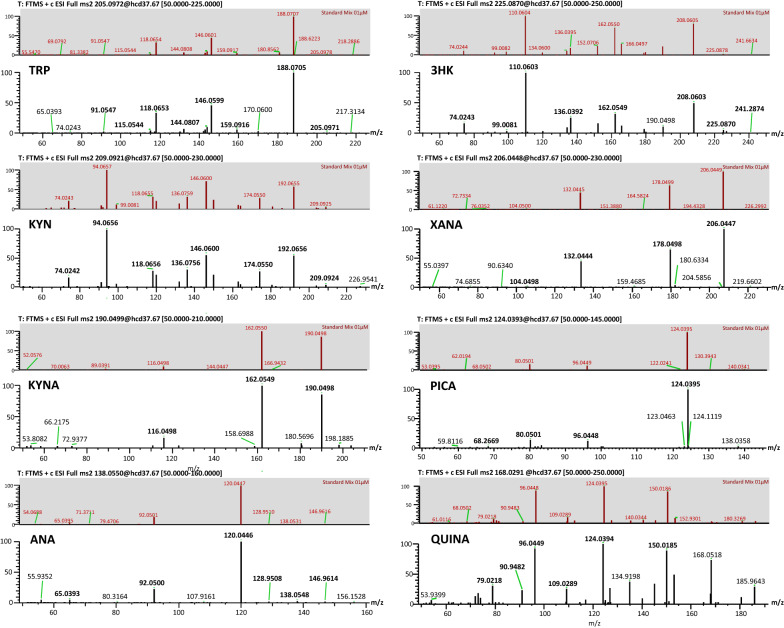
M^2^ mass spectra in positive ion mode for Tryptophan (Trp), kynurenine (KYN), kynurenic acid (KYNA), anthranilic acid (ANA), 3-hydroxy-kynurenine (3HK), xanthurenic acid (XANA), picolinic acid (PICA), and quinolinic acid (QUINA) in hemolymph compared with the corresponding M^2^ spectra of the standard mix (0.01 μM).

As the KP transcripts were successfully identified in all the tissues evaluated, we aimed to detect KP metabolites in LS hemolymph utilizing UHPLC-MS. Each target metabolite was revealed as a chromatographic peak, eluting at its specific retention time, using mass range chromatograms with theoretical m/z ± five ppm values of putative [M + H]^+^ molecular ions. For other identity confirmation, peak areas were used to compare each potential metabolite in hemolymph with those observed in hemolymph spiked with a mixture of standards (1 μM final concentration) (Fig. 6). Subsequently, the MS^2^ fragmentation behavior was compared with that of the standard mix using the Q-Exactive PRM mode (Fig. 7). TRP, KYN, KYNA, ANA, 3HK, XANA, PICA, and QUINA were accurately defined by comparing retention time, accurate mass, and MS^2^ data. With the sole exception of 3HANA (not shown), for each compound, we observed a single peak in hemolymph that was increased in the spiked sample (Fig. 6).

The maximum mass errors between the theoretical and measured ones were between  − 0.67/1.18 ppm (Supplementary Table S4).

In hemolymph, TRP eluted at the retention time of 11.42 min and showed characteristic product ions at m/z 188.0705, 146.0599, and 118.0653 matching the pattern of the reference standard.

KYN eluted in hemolymph at 8.05 min showed a protonated molecular ion at 209.0294 and product ions at 94.0656, 192.0656, 146.0600, 136.0756, like those of the standard mix.

Kynurenic acid had a retention time in the hemolymph of 12.64 min and yielded an m/z of 190.0499 as [M + H]^+^ ion and other fragmented ions at 162.0549 and 116.0498 similar to the pattern of the reference standard.

The fragmentation of ANA in hemolymph, eluted at 10.37 min, with ions at 120.0446 and 92.0500, matched the corresponding standard. In hemolymph, 3HK eluted at the retention time of 5.31 min and showed characteristic product ions at m/z 110.0603, 208.0603, 136.0392, and 162.0549 matching the pattern of the reference standard.

XANA eluted in hemolymph at 14.14 min showed a protonated molecular ion at 206.0447 and product ions at 178.0498 and 132.0444 like those of the standard mix.

In our samples, PICA and QUINA were eluted at 2.65 and 2.21 min, respectively, and both showed a fragmented pattern like that of their corresponding standard. PICA exhibited a m/z of 124.0395 as [M + H]^+^ ion and other fragmented ions at 96.0448 and 80.0501, while the fragmentation of QUINA provided several fragments at 150.0185, 124.0394, and 96.0449.

## Discussion

In this study, we identified transcripts of putative enzymes and metabolites of the KP in the pond snail *L. stagnalis* (LS), a promising candidate for translational neuroscience research ^20^.

The KP possesses a high level of conservation between vertebrates and invertebrates, is pharmacologically inducible, and is especially relevant to neuroscience ^4,29^. Under physiological conditions, the KP is the primary route for tryptophan catabolism and the starting point for synthesizing the ubiquitous co-enzyme nicotinamide adenine dinucleotide, which, in turn, fulfills the cellular energy requirements ^10^. As the KP plays a crucial role in immune function and energy metabolism, aberrations of its molecular cascade have been identified in many human diseases and disorders, including inflammation/immune disorders, endocrine/metabolic conditions, Alzheimer's disease, amyotrophic lateral sclerosis, Huntington's disease, cancer, depression, and schizophrenia ^10,30–32^.

The KP has been studied only in two invertebrate models: the worm *C. elegans* and the fly *D. melanogaster*. In *C. elegans*, alterations in the KP cause neurodegeneration and aging ^33^, whereas in *D. melanogaster* a correlation between elevated levels of 3-hydroxy-kynurenine and synaptic plasticity has been reported ^34^. To our knowledge, apart from these two studies, this is the first complete characterization of the KP in a molluscan model.

In the last few years, an increasing effort has been made to produce comprehensive *L. stagnalis* transcriptomic and proteomic databases ^21,35–38^. Still, along with the identification of conserved genes and transcripts, the lack of their functional and pharmacological characterization has limited molecular studies in this model to date.

Given this, instead of generating a new transcriptome, here we compared one of the latest assemblies of *L. stagnalis* genome with annotated proteins of *H. sapiens, M. musculus,* and *B. glabrata*; contigs were also combined with transcripts from the Sadamoto et al., (2012) dataset to provide an estimation of transcript abundance ^18^. Thanks to this approach, we identified contigs of *L. stagnalis* matching with the genes coding for the enzymes of the KP in the annotated genome of *B. glabrata*. All the enzymes were identified univocally, in *B. glabrata* the IDO-like orthologue is named myoglobin-like since it is possible that in molluscs it functions in their buccal mass as myoglobin, not as an IDO ^39^, and two putative isoforms of kynurenine aminotransferases, KYAT I-III and AADAT (KYAT II), were included in the analysis. In fact, human and rodent genomes encode four aminotransferases (KYAT I-IV) ^40^, KYAT I and KYAT III share the highest sequence identity and are phylogenetically distant from AADAT ^41^.

These contigs then matched univocally (except TDO) with putative transcripts in *L. stagnalis* CNS. All putative transcripts identified contained an ORF; the predicted protein had several amino acids very close to those of the human protein and the corresponding orthologue of several organisms extensively employed as a standard models in preclinical studies. Consistent with previous studies, our phylogenetic analyses revealed a high degree of sequence conservation between *L. stagnalis* and other gastropods and model organisms ^1,29^.

The transcripts predicted in silico by the computational analysis were validated using Sanger sequencing and the expression patterns of the KP enzymes were quantified in different tissues of *L. stagnalis*. Studies from mammals and humans demonstrated that the KP metabolites act as versatile mediators in inter-cell or inter-organ cross-talk, as they can be exchanged among tissues and be either metabolized or exert a biological effect ^1^. In particular, considerable evidence demonstrates a strong link between the KP, proinflammatory cytokines, and the nervous and immune systems ^30^. Moreover, some KP metabolites can be further processed, leading to the production of NAD^+^^32^, which plays a crucial role in energy production and is extremely important in organs like the brain and muscles ^42,43^. Furthermore, studies from rodents and humans, underlined the importance of the tryptophan metabolism through the KP in the gut-brain axis communication and the link between depression and bowel inflammation ^44^. Finally, the KP controls the immune and inflammatory environment of the epididymis in mice ^45,46^.

Given the importance of the KP in these body areas, in this study, we compared the mRNA expression levels in the *L. stagnalis* CNS, gut (i.e., digestive gland), stomach, muscle (i.e., foot), penis, and hemocytes (i.e., invertebrate immune system cell). To our knowledge, we provide the first evidence that the enzymes of the KP are widely expressed in the whole body of *L. stagnalis*, suggesting that the KP is active in various tissues in this organism as it does in mammals ^47^. Specifically, we found that Lym TDO-like is expressed mainly in the gut and muscle, this is consistent with data from vertebrates showing that the TDO enzyme is particularly active in hepatocytes, which are part of the alimentary system and the gut ^47,48^.

IDO has been identified in vertebrates, lower vertebrates, several invertebrates, fungi, and bacteria. In vertebrates were identified two distinct *IDO* paralogues, *IDO1* and *IDO2,* originated from a gene duplication event that possibly occurred before the divergence of vertebrates. Many of the isoforms of IDO enzyme identified in invertebrates ^5,49^ show low catalytic efficiency/affinity for TRP, similar to vertebrate IDO2s ^49^. Here we identify a single IDO transcript ubiquitously distributed in all tissues, these data are consistent with those from mammals, where IDO1 and IDO2 are widespread throughout the body and are expressed in almost every type of cell.

Furthermore, we found that Lym HAAO-like, Lym AADAT-like, and Lym KYAT-like are also mainly expressed in the gut. In rodents, KYAT-I and KYAT-III share similar expression patterns: their mRNA levels are much higher in the liver and neuroendocrine tissues than in the CNS ^40,50^. This trend is reported also in LS for Lym KYAT-like. AADAT expression is higher in the rodent brain where it is mostly located in astrocytes, while in the periphery it is detected as well in most tissues with the sole exception of the skeletal muscle. In *L. stagnalis* we demonstrated that its expression levels in the foot muscle are like those observed in the CNS.

Our results support a strong connection between the KP and the digestive gland of *L. stagnalis*. In mollusks this organ known also as hepatopancreas is involved, among others, in the secretion of digestive enzymes and food and nutrients absorption. From a translational point of view, this is in line with the established role proposed for KP in regulating intestinal (patho) physiology in humans and rodents. Alterations of the TRP metabolism can give rise to gastrointestinal dysfunction ^51^ and in turn the gut microbiota can influence the KP.

Consequently, changes in gut biodiversity may cause increased gut permeability and systemic and CNS inflammation ^52^. Thus, future studies may investigate whether similar results can be reproduced in *L. stagnalis* by changing the composition of the snails’ microbiota to further understand the complex communication between GI microbes and tryptophan catabolism and identify possible targets for the treatment of various human diseases.

To complete the biochemical characterization of the KP, we also performed UHPLC-Q Exactive Mass Spectrometry to identify KP metabolites in hemolymph and hemocytes of *L. stagnalis*. This analysis led to the identification of tryptophan, kynurenine, kynurenic acid, anthranilic acid, 3-hydroxy-kynurenine, xanthurenic acid, picolinic acid, and quinolinic acid.

In this context, studying the molecular and behavioral effects induced by the manipulation of the KP in *L. stagnalis* represents an appealing strategy both to assist the study of current unsolved questions and to drive the field forward. This model not only can help discover additional physiological and pathological roles for kynurenines but also may contribute to the development of new therapies based on these roles.

Finally, our results not only constitute new evidence on the potentialities of the pond snail *L. stagnalis* as a powerful model for neuroscience but also open numerous scenarios in the use of this model in translational neuroscience and the efficient study and characterization of other molecular pathways.

## Methods

### Identification of putative KP genes in *L. stagnalis* genome

To identify putative KP genes in *L. stagnalis*, we first downloaded its genome assembly in contigs from the NCBI Genome database (https://www.ncbi.nlm.nih.gov/genome/?term=Lymnea+stagnalis). Then, we used the program *BLASTx* of the NCBI BLAST + suite (release 2.2.28) to align all contigs to the protein sequences annotated with RefSeq IDs of i) *B. glabrata* (36,675 sequences), ii) *M. musculus* (76,216 protein sequences) and iii) *H. sapiens* (110,386 sequences). All protein sequences have been downloaded from https://ftp.ncbi.nih.gov/genomes/refseq/, and *BLASTx* results have been filtered, setting an e-value cutoff of 1E-6. Finally, starting from the RefSeq ID of each aligned protein sequence, we used the *queryMany* function of the *mygene* R package (https://www.bioconductor.org/packages/release/bioc/html/mygene.html) to annotate contigs with *B. glabrata*, mouse, and human gene symbols and with Gene Ontology (GO) and KEGG pathway terms.

To determine whether homologs of KP genes are indeed expressed transcripts in *L. stagnalis*, we downloaded the raw RNA-sequencing (RNA-seq) data of the *L. stagnalis* central nervous system from the NCBI SRA database (https://www.ncbi.nlm.nih.gov/sra/DRX001464) ^18^. We then assembled *L. stagnalis* transcriptome with Cufflinks (2.0.2; http://cole-trapnell-lab.github.io/cufflinks/), aligning RNA-seq reads to its genome with TopHat (2.1.0; https://ccb.jhu.edu/software/tophat/index.shtml). Before assembly, all reads have been trimmed and filtered for quality using Trimmomatic (http://www.usadellab.org/cms/?page=trimmomatic). Specifically, we trimmed the read tails when the read quality was lower than 20 and removed nine nucleotides from the start of each read (HEADCROP: 9 in Trimmomatic). The gene expression level of each contig was then quantified by converting the counts of uniquely mapping reads into TPM (Transcripts Per Million), as follows:

$$\user2{ }TPM_{i} = \frac{{X_{i} }}{{l_{i} }} \cdot \left( {\frac{1}{{\mathop \sum \nolimits_{j} \left( {\frac{{X_{j} }}{{l_{j} }}} \right)}}} \right) \cdot 10^{6}$$

where *X*_*i*_and *l*_*i*_ are the read count and the length of contig *i*, respectively.

To identify the exact sequence of transcripts annotated to the kynurenine pathway, we manually extracted putative exonic sequences visualizing RNA-seq reads on the *L. stagnalis* genome with Integrative Genomics Viewer (IGV, http://www.broadinstitute.org/igv/). Then, we aligned these sequences to the transcriptome shotgun assembly of *L. stagnalis* using *BLASTn* to retrieve the precise mRNA sequence.

### Sequence analysis

We predicted the amino acid sequences using the Open Reading Frame (ORF) Finder tool of NCBI (https://www.ncbi.nlm.nih.gov/orffinder/). Alignments of nucleotide and translated protein sequences were performed with http://nadv.herokuapp.com/. ORF sequences were fed into the protein family database PFAM (https://pfam.xfam.org/) to identify the major conserved characteristic domains belonging to each previously organized sequence group ^53^.

### Phylogenetic analysis

To determine sequence evolutionary organization and distribution, as well as to provide further evidence of the presence of putative proteins in *L. stagnalis*, we performed a phylogenetic analysis using the UPGMA method ^54^ of Molecular Evolutionary Genetic Analysis (MEGA)-X tool (https://www.megasoftware.net/). Specifically, we used the amino acid sequences of deuterostomes and protostomes organisms such as *H. sapiens*, *M. musculus, R. norvegicus, C. elegans,* and mollusks such as *A. californica* and *B. glabrata*, obtained from NCBI. The evolutionary distances were computed using the Poisson correction method and are in the units of the number of amino acid substitutions per site. The predicted amino acid sequences of *L. stagnalis* KP enzymes were aligned with those of *H. sapiens*, *M. musculus, R. norvegicus, C. elegans, A. californica*, and *B. glabrata* molluscan using T-Coffee tool (http://tcoffee.crg.cat/apps/tcoffee/do:regular) ^55^ and visualized with BoxShade (v3.21; https://bio.tools/BoxShade).

### Ethics statement

Pond snails *L. stagnalis* are abundant on earth northern hemisphere and are not endangered or a protected species. Experiments on pond snails are not subject to the approval of our ethics committee. Nonetheless, every effort was made to maximize the well-being of the snails during the behavioral procedures.

### Snails and colony maintenance

Laboratory-reared *L. stagnalis*, originally derived from a stock generously donated by the Vrije University in Amsterdam (The Netherlands), were used in this study. Animals were maintained in aquaria at the University of Modena, and Reggio Emilia (Italy) at 21–23 °C in well-aerated dechlorinated tap water on a 12/12 h light/dark cycle (lights on at 08:00 a.m.). Six-month-old snails having shell lengths of 20–25 mm were used in these experiments. Animals were fed pesticide-free lettuce twice a week.

### Hemolymph collection

The mechanical hemolymph collection was carried out by tickling the foot sole of the snail with the tip of a micropipette; as a reflex mechanism, the hemolymph is extruded through the hemal pore. The hemolymph was then gently collected in polypropylene tubes and stored at  − 80 °C before analysis.

### RNA extraction and retrotranscription

Following hemolymph collection, animals were anesthetized on ice for 10 min, and the central ring ganglia (buccal ganglia were excluded), digestive gland, stomach, foot muscle, and penis were dissected and stored at -80 °C before analysis. Hemocytes were isolated by centrifuging 500 μL of hemolymph for 10 min at 1,000 × g at 4 °C. The supernatant was discarded, and the pellet containing hemocytes was resuspended with 1 mL of Trizol (Merck KGaA; Darmstadt, Germany). Total RNA extraction and DNAse treatment were performed on four replicates of a pool of 4 animals using GenElute Total RNA Miniprep Kit and DNASE70-On-Column DNase I Digestion Set (Merck KGaA; Darmstadt, Germany) as previously described ^56,57^. Four hundred ng of total RNA was reverse transcribed with a High-Capacity cDNA Reverse Transcription Kit (Life Technologies Corporation) in 20 µL of the reaction mix.

### Qualitative PCR analysis and sequencing

Qualitative PCR was performed using Dream Taq DNA polymerase (Thermo Scientific, Waltham, Massachusetts, United States) under the general 3-step amplification of 95 °C for 5 min, followed by 35 cycles of 95 °C for 30 s, 55 °C for 30 s; 72 °C for 30 s, and final extension of 72 °C for 7 min. Primer sequences were designed by NCBI Primer-BLAST software (https://www.ncbi.nlm.nih.gov/tools/primer-blast/) and were synthesized by Merck KGaA (Darmstadt, Germany). PCR products were electrophoresed on agarose gel (2%), and DNA fragments were visualized by UV illumination to confirm the correct amplicon size. PCR products (600–800 bp) were purified using the High Pure PCR Product Purification Kit (Roche Diagnostics Corporation, USA) following the manufacturer’s instructions and were directly sequenced using the Sanger sequencing method. Sequencing was performed using ABI PRISM 3130xl Genetic Analyzer (Applied Biosystems, California, USA) and BigDye Terminator v1.1 Cycle Sequencing Kit (Life Technologies Corporation, Massachusetts, USA). The sequence analysis of the PCR fragments was performed using Sequence Scanner Software 2.0 (Applied Biosystems, California, USA), and sequences were compared with the contigs of *L. stagnalis* using the online version of *BLASTn* (https://blast.ncbi.nlm.nih.gov/Blast.cgi?PROGRAM=blastn&PAGE_TYPE=BlastSearch&BLAST_SPEC=&LINK_LOC=blasttab&LAST_PAGE=blastn).

### Design, validation, and optimization of primers for quantitative PCR analysis

Candidate primers for quantitative Real-Time PCR (qRT-PCR) were designed with NCBI Primer-BLAST software (https://www.ncbi.nlm.nih.gov/tools/primer-blast/) and synthesized by Merck KGaA (Darmstadt, Germany). Primers were designed to have a length of 19–23 nucleotides, a melting temperature between 58 and 62 °C, a GC content between 40 and 60%, and generating an amplicon between 100 and 200 bp. First, primer specificity was assessed by qualitative PCR as previously described; each experiment contained two biological replicates of cDNA from central ring ganglia of *L. stagnalis* and *P. canaliculata* (kindly provided by Prof. Davide Malagoli, UNIMORE), minus reverse transcription (RT) controls to assess the genomic DNA, and non-template controls. PCR products were run as previously described. Then primer efficiency was evaluated in Bio-Rad CFX Connect. Curves were generated from four-fold serial dilutions of cDNA run in triplicate. Amplification of all genes was detected with SyBR Green dye which generates fluorescence based on the synthesis of double-stranded DNA. The reactions contained 5 µL of cDNA with 10 µL of Bio-Rad SsoAdvanced Universal SyBR Mix, 300 nM forward and reverse primer concentration, and topped to 20 µL with ddH_2_O. Each point was run in triplicate. The qPCR reactions occurred in a Bio-Rad CFX Connect thermocycler running a custom program. The custom qPCR program consisted of 95 °C for 30 s, 40 cycles of 95 °C for 15 s, and 60 °C for 30 s. The machine read the plate to measure fluorescence at the end of each cycle.

### Statistical analysis

Stability values were calculated for each candidate housekeeping gene elongation factor 1α– Lym EF1α and β–tubulin – Lym βTUB) using NormFinder (https://moma.dk/normfinder-software) ^58^, taking into account intra and intergroup variation. The geometric mean of Cqs of the reference genes was used as calibrator. For an appropriate application of the comparative ΔΔCt method, it was demonstrated that the amplification efficiency of the target genes and endogenous control gene were approximately equal**.** Extreme outliers were excluded before the statistical analysis using the boxplot tool in SPSS (more than 3 × the interquartile range outside the end of the interquartile box). Analyses were conducted using SPSS for Windows v.28 (SPSS Inc., Chicago, USA).

### Chemicals and materials

LC–MS grade acetonitrile and formic acid were purchased from Merck. Deionized water was obtained from MilliQ IQ 7000 system (Merck Millipore). Tryptophan (Trp), kynurenine (KYN), kynurenic acid (KYNA), anthranilic acid (ANA), 3-hydroxy-kynurenine (3HK), xanthurenic acid (XANA), picolinic acid (PICA), and quinolinic acid (QUINA) were of analytical grade (Merck KGaA (Darmstadt, Germany)).

### Hemolymph preparation for metabolite analysis

Two hundred microliters of hemolymph were dounce homogenized to disrupt hemocytes, then KP metabolites were extracted as previously described ^59^: an equal volume of ice-cold 1 M perchloric acid (Merck KGaA) fortified with deuterated tryptophan, kynurenine, kynurenic acid and quinolinic acid (Buchem B.V., Apeldoorn, The Netherlands) as internal standard (final concentration 1 µM) was added to the homogenized sample that was then centrifuged (15,000 × *g*, 10 min at 4 °C) and the supernatants were collected for direct injection into the LC–MS/MS.

### Instrument (UHPLC-Q exactive mass spectrometer) conditions

The analyses of KP metabolites were performed using ultra-high-performance liquid chromatography (UHPLC) coupled with mass spectrometry using heated electrospray ionization (HESI) in positive ionization mode. Chromatographic separations were carried out on a UHPLC Ultimate 3000 system (Thermo Electron Corporation, San Jose, CA, United States) equipped with an UltiMate 3000 HPG-3400RS High Pressure Mixing Biocompatible Gradient Pump with a Discovery HS F5 HPLC Column (Merck KGaA) (L × I.D. 25 cm × 4.6 mm, 5 μm) thermostated at 25 °C. The mobile phase comprised 0.1% formic acid in water (eluent A) and acetonitrile (eluent B). The gradient elution program was set as follows: 0–0.2 min 2% B; 0.2–18.2 min 38% B; 18.2–24.9 min 98% B; finally, the column was equilibrated with 2% B for 12 min. The flow rate was 1.5 mL/min. The sampler (UltiMate 3000 WPS-3000TRS) was kept at 15 °C. The injection volume was 40 µL. High-resolution MS and MS^2^ spectra were obtained on a Q Exactive Hybrid Quadrupole-Orbitrap Mass Spectrometer (Thermo Electron Corporation, San Jose, CA, United States); HESI optimized parameters were set as follows: sheath gas flow rate, 37 arbitrary unit; auxiliary gas flow rate, 28 arbitrary unit; sweep gas flow rate, 2 arbitrary unit; spray voltage, 3.20 kV; capillary temperature, 320 °C; S-lens RF level, 55.0; aux gas heater temperature, 290 °C. Targeted selected ion monitoring (tSIM) acquisition was performed using an inclusion list (based on *m*/*z* of each target and the expected retention time; Supplementary Table X) at a mass resolution of 70,000, an isolation window of 1.0 m/z, AGC target of 2e5, and Maximum IT of 247 ms. Parallel reaction monitoring (PRM) mode was carried out at a mass resolution of 17,500, an isolation window of 1.0 m/z, AGC target of 5e5, Maximum IT of 120 ms, normalized collisional energies (NCE) of 15, 28, 70 were employed for fragmentation. The mass spectrum of each metabolite was obtained by injecting a standard mix at a concentration of 0.01 μM.

Raw data were acquired and processed using the Xcalibur software (Version 4.2.4, Thermo Electron Corporation, San Jose, CA, United States).

## Supplementary Information

Supplementary Information 1.

Supplementary Information 2.

## Data Availability

The datasets generated during and/or analyzed during the current study are available from the corresponding author on reasonable request.
