# Supplementary material for: Identification and characterization of the kynurenine pathway in the pond snail Lymnaea stagnalis
Source: Sci Rep. 2022 Sep 16;12:15617. doi: 10.1038/s41598-022-19652-0 (PMC9481534; doi:10.1038/s41598-022-19652-0)

Supplementary Material

Supplementary table 1: Included separately as **Supplementary Dataset File**

Supplementary Table S2. Putative genes coding for the KP enzymes in *L. stagnalis.* For each contig, are reported the ID, the contig length (bp), the FX_ value corresponding to *L. stagnalis* TSA, and the RefSeq protein ID identified in *B. glabrata* with the corresponding gene definition. When available the RefSeq protein ID identified in *M. musculus* and *H. sapiens* are also reported, together with the gene symbol.

Lymnaea stagnalis				Homology association by BlastX						
Contigs			Transcriptome	*Biomphalaria glabrata*			*Mus musculus*		*Homo sapiens*	
Contig ID	Contig length (bp)	RNAseq TPM value	FX_TSA	RefSeq protein ID	Gene symbol	GENE definition	RefSeq protein ID	Gene symbol	RefSeq protein ID	Gene symbol
FCFB01037212.1	19708	0.211	FX190660.1	XP_013068349, XP_013068353, XP_013068352, XP_013068350, XP_013068354, XP_013068351, XP_013068348	LOC106056257	**myoglobin-like**	NA	NA	NA	NA
FCFB01037213.1	10282	2.481		XP_013068350, XP_013068348, XP_013068351, XP_013068349, XP_013068354, XP_013068353, XP_013068352			NA	NA	NA	NA
FCFB01037214.1	5124	4.716		XP_013068348, XP_013068349, XP_013068351, XP_013068353, XP_013068352, XP_013068350			NA	NA	NA	NA
FCFB01109750.1	7853	0.726	FX206683.1	XP_013068953, XP_013068952, XP_013068951, XP_013068954	LOC106056678	**tryptophan 2,3-dioxygenase-like**	NA	NA	NA	NA
FCFB01109751.1	5146	0.324	FX225637.1	XP_013068954, XP_013068952, XP_013068951, XP_013068953			NA	NA	NA	NA
FCFB01255639.1	922	1.112	FX224560.1	XP_013068951, XP_013068954, XP_013068952, XP_013068953			NA	NA	NA	NA
FCFB01077058.1	4544	0.621	FX191423	XP_013073423	LOC106060173	**kynurenine formamidase-like**	NA	NA	NA	NA
FCFB01161380.1	3637	1.745		XP_013073423			NA	NA	NA	NA
FCFB01232167.1	1538	1.084		XP_013073423			NA	NA	NA	NA
FCFB01020652.1	28977	15.087	FX185910.1	XP_013064773	LOC106053720	**kynurenine 3-monooxygenase-like**	NP_598570	Kmo	NP_003670, XP_016858139, XP_005273394, XP_016858138, XP_005273395	KMO
FCFB01033100.1	41166	4.438	FX191915.1	XP_013067258	LOC106055518	**kynurenine--oxoglutarate transaminase 1/3-like**	NP_001280489, XP_006501494, XP_011238416, NP_776124	Kyat3	NP_001008662	KYAT3
FCFB01016053.1	18986	8.370	FX183988.1	XP_013087071	LOC106071491	**2-aminoadipate transaminase-like**	NA	NA	NA	NA
FCFB01070793.1	13125	2.085	FX188572.1	XP_013068429	LOC106056296	**kynureninase-like**	XP_011237480, NP_001276522, XP_011237481	Kynu	NP_003928, XP_011510404, NP_001186170	KYNU
FCFB01141163.1	10604	2.545	FX195327.1	XP_013070925	LOC106058097	**3-hydroxyanthranilate 3,4-dioxygenase-like**	NA	NA	NA	NA
FCFB01269750.1	775	0.331		XP_013070925			NA	NA	NA	NA
FCFB01196791.1	649	0.593	FX187039.1	XP_013081879, XP_013081878	LOC106067265	**2-amino-3-carboxymuconate-6-semialdehyde decarboxylase-like**	NP_001028213	Acmsd	NP_612199	ACMSD
FCFB01209656.1	3764	1.976		XP_013081879, XP_013081878			NP_001028213	Acmsd	XP_005263647	ACMSD
FCFB01252153.1	971	16.042		XP_013081878, XP_013081879			NP_001028213	Acmsd	NP_001294912, XP_016858814, XP_016858815, XP_005263646, XP_005263645, XP_011508894	ACMSD
FCFB01272611.1	752	4.688		XP_013081879, XP_013081878			NP_001028213	Acmsd	XP_005263646, NP_001294912, XP_011508894, XP_016858815, XP_016858814, XP_005263647, XP_005263645	ACMSD
FCFB01319919.1	524	6.973		XP_013081879, XP_013081878			NP_001028213	Acmsd	XP_005263646, NP_001294912, XP_005263647, XP_005263645, XP_011508894, XP_016858815, XP_016858814	ACMSD

Supplementary Figure S1. Validated primers for sequencing. For each putative enzyme of the KP, are reported the relative transcript FX_, and the position of the forward (FW) and reverse (RV) primers.

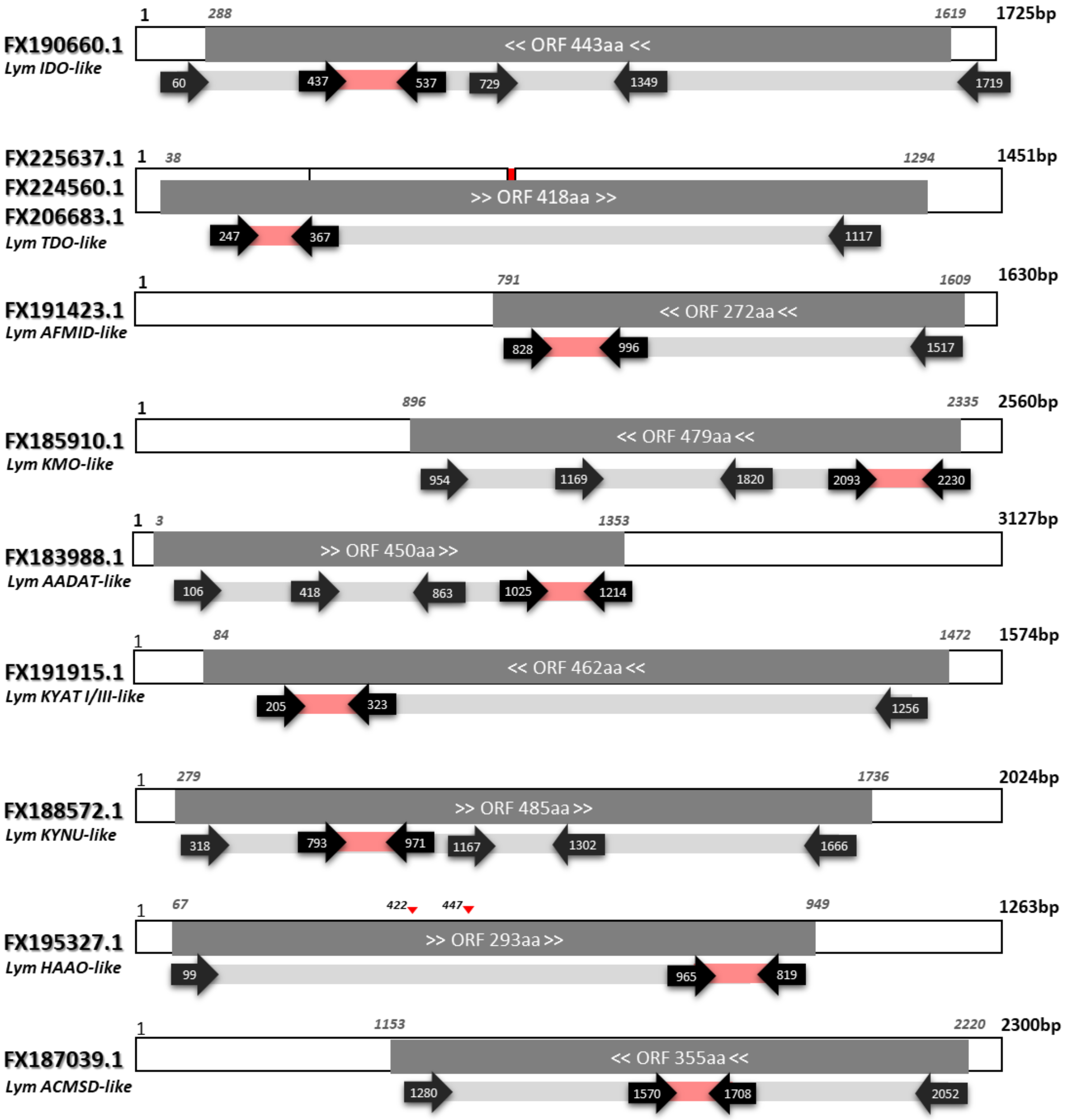

Supplementary Figure S2. Identification and location of the conserved domain for each putative enzyme of the KP in *L. stagnalis*. (A) Lym IDO-like, (B) Lym TDO-like, (C) Lym AFMID-like, (D) Lym KMO-like, (E) Lym AADAT-like, (F) Lym KYAT I/III-like, (G) Lym KYNU-like, (H) Lym HAAO-like, (I) Lym ACMSD-like.

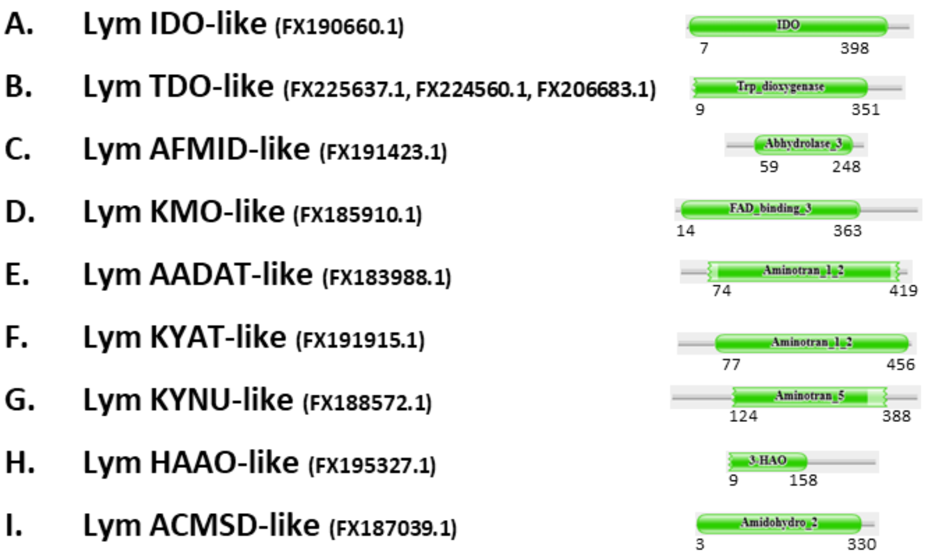

Supplementary Table S3. Validated primers for sequencing and gene expression analysis. For each predicted enzyme of the KP and the housekeeping genes (Lym EF1a and LymbTUB) are reported the relative transcript FX_, the forward (FW) and reverse (RV) primers' sequences, with the corresponding position on the gene.

MATCH ON	PREDICTED ENZYME		POSITION ON TRANSCRIPT	SEQUENCE
FX190660.1	**Lym IDO-like** Predicted *Lymnaea stagnalis* indoleamine 2,3-dioxygenase (IDO)	FW	60	CGGTTCTAACATAATTCCTC
		FW	437	ACCTGATGTCTTTCAAAAAT
		RV	537	TATTCAAGCTTCACAAACTG
		FW	729	AAAAGCTCTTTTGTCTTCAC
		RV	1349	GATAAAGGTGTACCACAGGT
		RV	1719	GCGATACAACAAAAATACAA
FX225637.1	**Lym TDO-like** Predicted *Lymnaea stagnalis* tryptophan 2,3 dioxygenase (TDO)	FW	247	TGTCAGAGATATGTTCATGC
FX225637.1		RV	367	CTCTAGAATGTCGATTTGGT
FX206683.1		RV	1117	CAGTGGTGGTATGTAGTCAC
FX191423.1	**Lym AFMID-like** Predicted *Lymnaea stagnalis* kynurenine formamidase(AFMID)	FW	828	ACAAAATTAGGTTCCGTAAG
		RV	966	ATGATCCTCCAGAGTTTAGA
		RV	1517	AAGTTACGGAGACTCAGAGA
FX185910.1	**Lym KMO-like** Predicted *Lymnaea stagnalis* kynurenine 3-monooxygenase (KMO)	FW	954	GGAGTTTCTAGATTCCAATG
		FW	1169	ATTGTCCAATTTCTTCCTAA
		RV	1820	GGCTTACTCCTCTGTTAGAA
		FW	2093	TTTTGTGATGGTGTCTTCTA
		RV	2230	TTCAAGGTTGATTTGTATGA
FX183988.1	**Lym AADAT-like** Predicted *Lymnaea stagnalis* kynurenine/alpha-aminoadipate aminotransferase II (AADAT)	FW	106	ACTTGCTTATGAAATGGAGT
		FW	418	TCGTAGAAGATCCCAGTTAT
		RV	863	GGGAGCAAATATTTTAGAGA
		FW	1025	GTCAGCCTATAAGGAAAGAA
		RV	1214	GTAAACACCTGGAAGAAAAC
FX191915.1	**Lym KYAT I-III-like** Predicted *Lymnaea stagnalis* kynurenine oxoglutarate transaminase 1-3 (KYAT I-III)	FW	208	GGAGGTATCACTGTCAATTT
		RV	323	TACTTCATGATGGCTGATTA
		RV	1256	TGATATATCCAAACTTGCAG
FX188572.1	**Lym KYNU-like** Predicted *Lymnaea stagnalis* kynureninase (KYNU)	FW	318	ACTACACCTTTGCAAGAACT
		FW	793	TTCCTTCAGATCATTACACA
		RV	971	CTGTACACCAGAAAAACAAA
		FW	1167	ATTCATGAGAACTTCCAGAC
		RV	1302	GTGTGTTGCTGATTCTGTAG
		RV	1666	ATGAATCTGTGAACATCCTC
FX195327.1	**Lym HAAO-like** Predicted *Lymnaea stagnalis* 3-hydroxyanthranilate 3,4-dioxygenase (HAAO)	FW	99	TGACAGTTGGATAGATGAAA
		FW	695	GTCCAACTCTTTGGAGATAA
		RV	819	TGAGTAACAACTGACTCACC
FX187039.1	**Lym ACMDS-like** Predicted *Lymnaea stagnalis* 2-amino-3-carboxymuconate-6-semialdehyde decarboxylase (ACMSD)	FW	1280	TCAGGACTAAATTCTTCCAT
		FW	1570	TTTAGGAAATCTCTCAAGGA
		RV	1708	AAGAGCACAACTGTGTGATA
		RV	2052	GAAGCACGAATTAAAGAAAT
DQ278441.1	**Lym EF1α** *Lymnaea stagnalis* elongation factor-1α	FW	7	GTGTAAGCAGCCCTCGAACT
		RV	157	TTCGCTCATCAATACCACCA
X15542.1	**Lym βTUB** Snail mRNA fragment for β-tubulin	FW	92	GAAATAGCACCGCCATCC
		RV	219	CGCCTCTGTGAACTCCATCT

Supplementary Table S4. Validated primers for gene expression analysis. For each enzyme of the KP, are reported the relative transcript FX_, the forward (FW) and reverse (RV) primers' sequences, with the corresponding size (bp), efficiency, R^2 score resulting from the validation experiments, and the Ct value obtained with 20 ng of cDNA.

TARGET	**Transcript**	**PRIMER FW SEQUENCE**	**PRIMER RV SEQUENCE**	**SIZE (bp)**	**EFFICIENCY**	**R^2**	**Ct value 20 ng**
Lym IDO-like	FX190660.1	ACTTAGGAAGAGTTTCAGCA	TTAAACCTAATCCCACAGAC	179 bp	115.66%	0.981	25.41
Lym TDO-like	FX225637.1	CTCTAGAATGTCGATTTGGT	TGTCAGAGATATGTTCATGC	120 bp	119.89%	0.994	30.02
Lym-AFMID-like	FX191423.1	ACAAAATTAGGTTCCGTAAG	ATGATCCTCCAGAGTTTAGA	138 bp	114.07%	0.990	26.86
Lym-KMO-like	FX185910	TTTTGTGATGGTGTCTTCTA	TTCAAGGTTGATTTGTATGA	137 bp	94.82%	0.996	25.84
Lym-AADAT-like	FX183988.1	GTCCAACTCTTTGGAGATAA	TGAGTAACAACTGACTCACC	189 bp	119.37%	0.995	28.33
Lym-KYAT I/III-like	FX191915.1	GGAGGTATCACTGTCAATTT	TACTTCATGATGGCTGATTA	118 bp	125.30%	0.995	27.41
Lym-KYNU-like	FX188572.1	TTCCTTCAGATCATTACACA	CTGTACACCAGAAAAACAAA	178 bp	114.89%	0.991	27.34
Lym-HAAO-like	FX195327.1	CTCTAGAATGTCGATTTGGT	TGTCAGAGATATGTTCATGC	124 bp	117.46%	0.988	28.53
Lym-ACMSD-like	FX187039.1	TTTAGGAAATCTCTCAAGGA	AAGAGCACAACTGTGTGATA	138 bp	119.56%	0.996	26.82

Supplementary Figure S3. Species-specificity of the primers. For each primer, the amplification was observed only in the cDNA from *L. stagnalis* but not in the cDNA of another pulmonated gastropod, *P. canaliculata,* nor in the negative control (NCT), nor the control lacking the reverse transcriptase in the RT-PCR (RT-). Representative cropped images of the electrophoretic gels are reported (agarose 2%.), original images are reported in **Supplementary Figure S5**

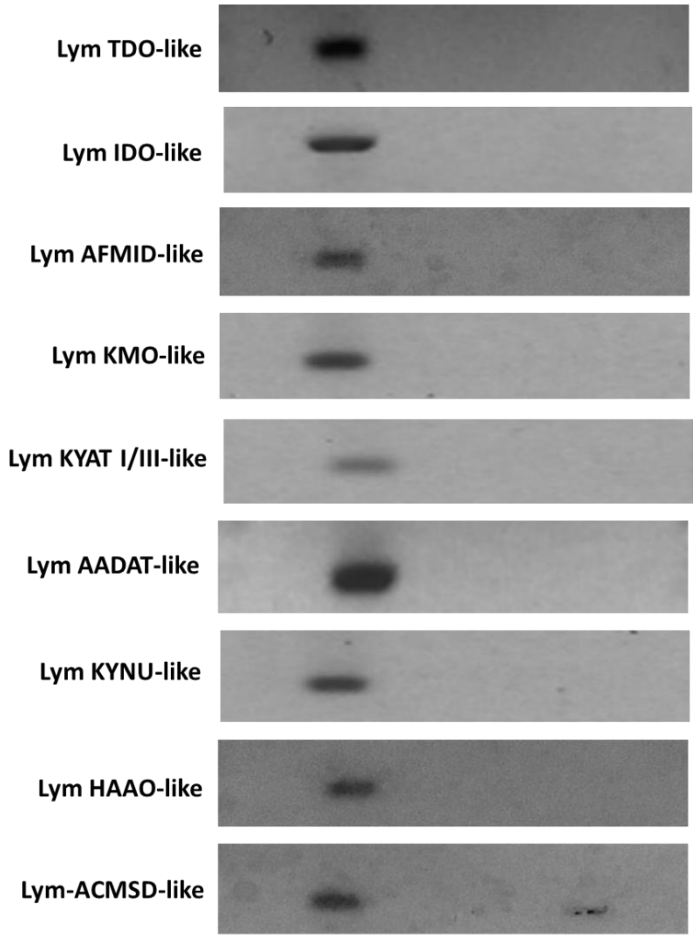

Supplementary Figure S4. Melting curves analysis. For each target, a single sharp peak with no primer-dimer was observed.

Melting curve analysis

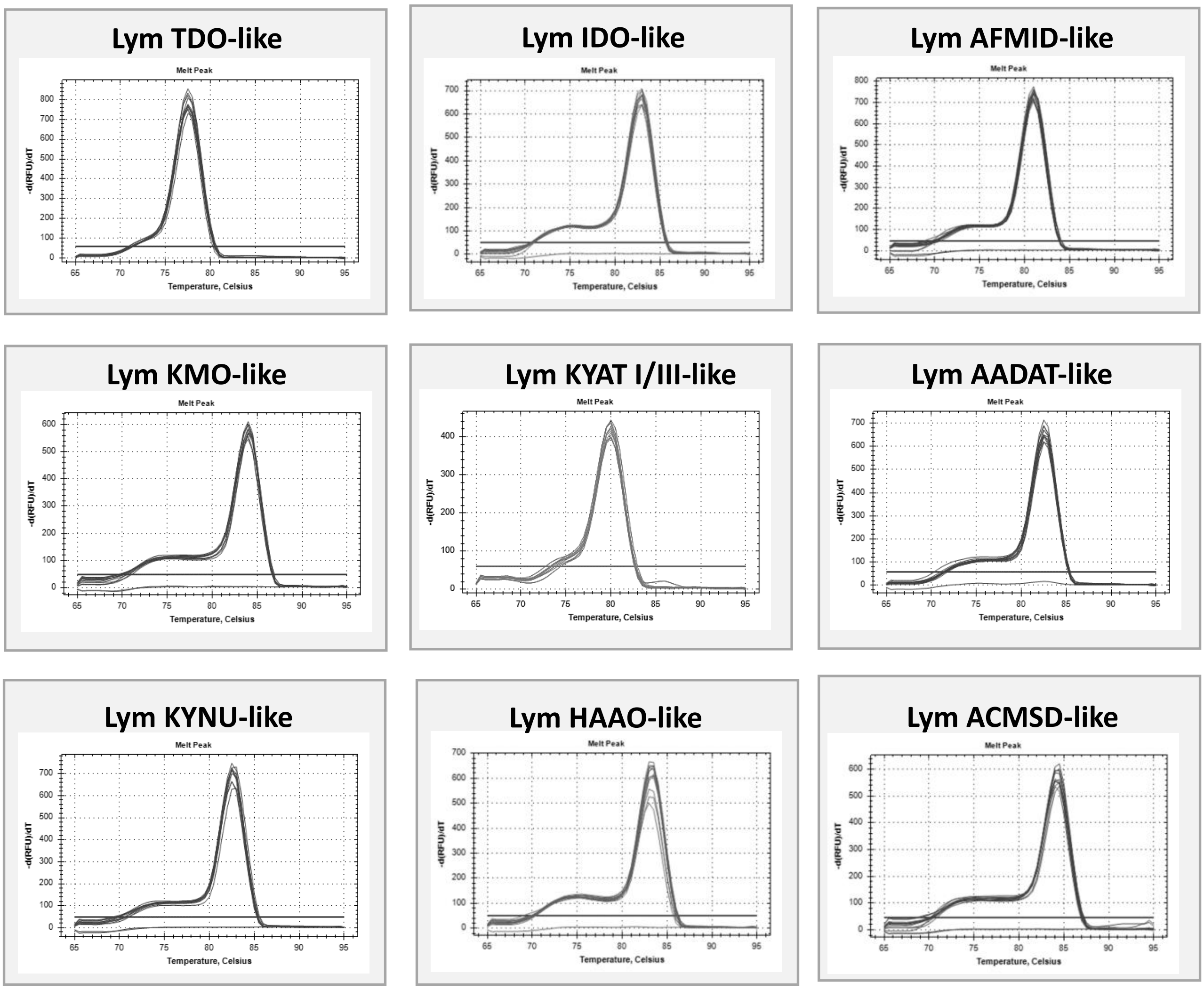

Supplementary Table S4. Characterization of KP metabolites in *L. stagnalis* hemolymph by UHPLC-Q Exactive mass spectrometer. RT: retention time; Theo m/z: theoretical mass charge ratio; Meas. m/z: measured mass charge ratio: ppm: parts per million.

Target	Formula [M]	RT [min]	Theo. m/z $[M+H]^+$	Meas. m/z $[M+H]^+$	Error [ppm]	Acquisition window [min]	Scan filter
**TRP**	$C_{11}H_{12}N_2O_2$	11.42	205.0972	205.0973	0.59	10.50-12.50	[204.5972-205.5972]
**KYN**	$C_{10}H_{12}N_2O_3$	8.05	209.0921	209.0919	-0.64	7.20-9.00	[208.5921-209.5921]
**KYNA**	$C_{10}H_7NO_3$	12.64	190.0499	190.0498	-0.27	11.80-16.00	[189.5499-190.5499]
**ANA**	$C_7H_7NO_2$	10.37	138.0550	138.0550	0.27	9.60-11.50	[137.5550-138.5550]
**3HK**	$C_{10}H_{12}N_2O_4$	5.31	225.0870	225.0868	-0.65	4.40-6.30	[224.5870-225.5870]
**XANA**	$C_{10}H_7NO_4$	14.14	206.0448	206.0447	-0.44	13.00-17.00	[205.5448-206.5448]
**PICA**	$C_6H_5NO_2$	2.65	124.0393	124.0395	1.18	1.40-3.30	[123.5393-124.5393]
**QUINA**	$C_7H_5NO_4$	2.21	168.0291	168.0290	-0.67	1.80-3.40	[167.5291-168.5291]

Supplementary Figure S5: Uncropped, full-length images (multiple exposures) of the electrophoretic gel reported in supplementary figure S3, MK:marker, LS: *L. stagnalis*, PC: *P. canaliculata*, NTC: negative control, RT-: control lacking the reverse transcriptase in the retrotranscription.

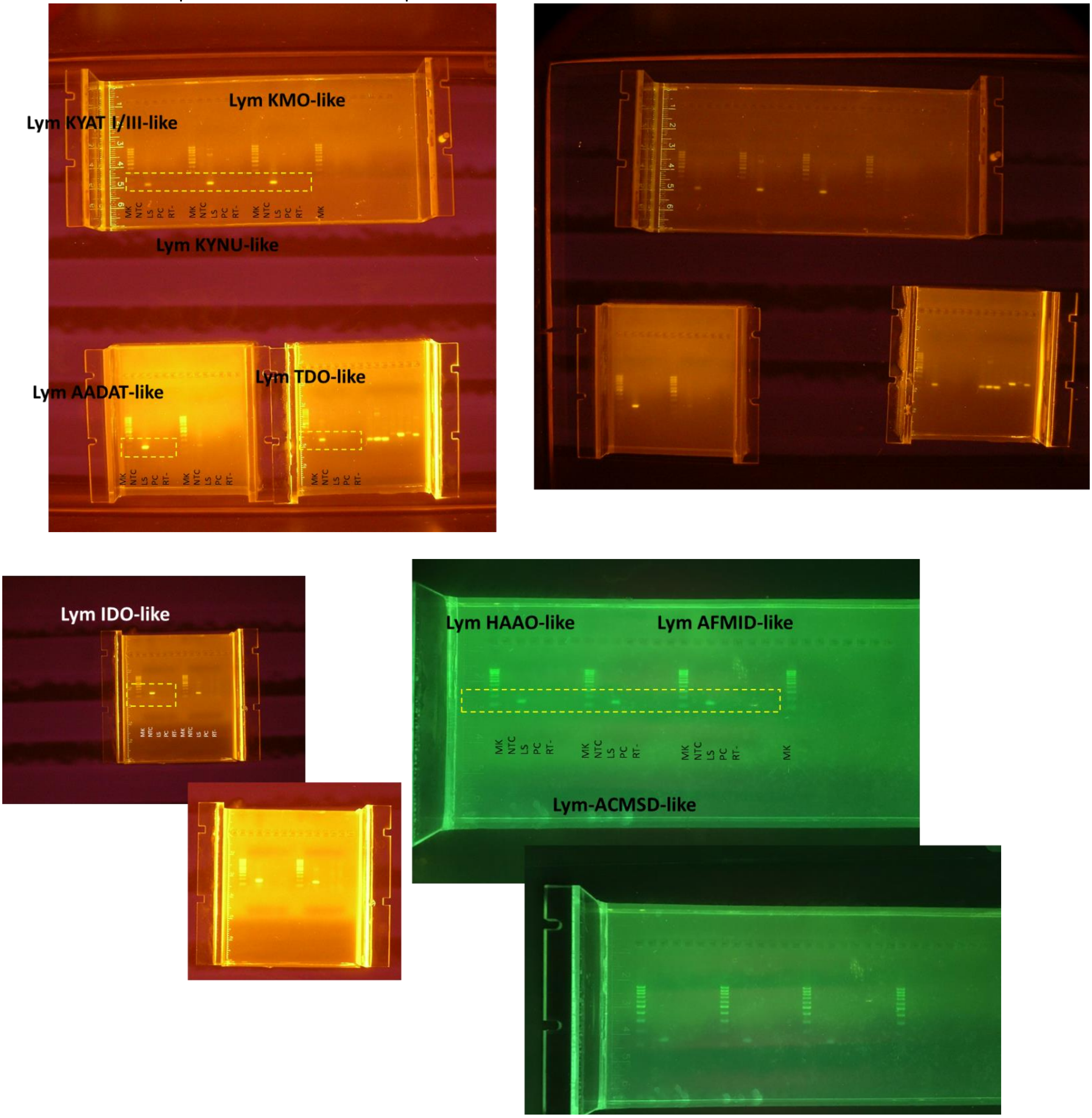

Supplement: Supplementary file 1 — Supplementary Information 1. [file 41598_2022_19652_MOESM1_ESM.pdf]
